# Continuous Laser Versus Micropulse Laser in the Treatment of Central Serous Chorioretinopathy: A Retrospective Study

**DOI:** 10.7759/cureus.53799

**Published:** 2024-02-07

**Authors:** Mihai Bica, Alexandru David, Florian Balta, Andrei Iacob

**Affiliations:** 1 Ophthalmology, Spitalul Clinic de Urgente Oftalmologice Bucuresti, Bucharest, ROU; 2 Ophthalmology, Clinica Retina, Bucharest, ROU

**Keywords:** visual acuity, subretinal fluid, subfoveal choroidal thickness, macular thickness, central serous chorioretinopathy, micropulse laser, continuous laser

## Abstract

Background: Central serous chorioretinopathy (CSCR) is a retinal disorder characterized by complex mechanisms leading to abnormal fluid accumulation under the retina. While management remains controversial, laser therapy has been successfully used. This study compares the efficacy of continuous laser (CL) and micropulse laser (ML) therapy in treating CSCR, focusing on reduction in macular thickness and improvement in visual acuity.

Methods: A retrospective cohort study was conducted, including patients with CSCR treated with either CL or ML. The primary outcome measured was the reduction in average macular thickness (AMT), alongside secondary outcomes like changes in best corrected visual acuity (BCVA), subfoveal choroidal thickness (SFCT), and resolution of subretinal fluid (SRF).

Results: The study evaluated 121 patients with CSCR, treated either with CL or ML. The primary outcome showed that the mean reduction in AMT was 51.14 µm (±20) in the CL group and 29.88 µm (±22) in the ML group, without a statistically significant difference (p=0.10). For the secondary outcomes, the improvement in BCVA was comparable in both groups, with CL at 0.15 (±0.1) and ML at 0.12 (±0.1) and no significant difference (p=0.41). However, in SFCT reduction, CL showed greater efficacy with a mean reduction of 32.19 µm (±15) compared to ML's 4.85 µm (±18), which was statistically significant (p=0.0004). The degree of SRF resolution showed no significant difference between the treatments (p=0.065).

Conclusions: Both CL and ML are effective in the management of CSCR, with CL being more effective in reducing SFCT. These findings suggest the need for personalized treatment strategies based on individual patient characteristics and underline the complexity of CSCR management.

## Introduction

Central serous chorioretinopathy (CSCR) is a relatively common retinal disorder that primarily affects young and middle-aged adults, with a higher prevalence in males than females [1]. The hallmark of CSCR is the accumulation of subretinal fluid (SRF) in the macular area, leading to symptoms such as a decrease in visual acuity and metamorphopsia [2]. While the complete pathogenesis of CSCR remains yet to be uncovered, certain factors have been involved, such as choroidal hyperpermeability, retinal pigment epithelium (RPE) dysfunction, and neurosensory detachment [3]. Several predisposing factors have also been identified, such as psychological stress, corticosteroid treatment, pregnancy, arterial hypertension, and type A personality, which represent notable associations [4].

The natural evolution of CSCR is usually self-limited with spontaneous absorption of SRF and recovery of visual acuity in 3-6 months [5]. However, in some cases, the disease may be refractory, or patients may experience episodes of recurrence, leading to chronic CSCR, eventually resulting in irreversible loss of photoreceptors and RPE, and therefore permanent loss of vision [6]. Management of CSCR remains controversial as there is no current consensus on the optimum timing, modality, or efficacy of intervention. The main objective of treatment is to accelerate recovery by facilitating SRF absorption, as well as to prevent recurrence and maintain good visual outcomes [7].

From the breadth of treatment modalities, laser treatment is one of the most utilized and studied options in the management of CSCR. Laser therapy aims to create focal or diffuse lesions at the level of the RPE-choroid complex, in order to reduce fluid outflow and re-establish the integrity of the outer blood-retinal barrier [8]. However, different types of laser therapy have different mechanisms, results, and complications, and thus, the choice between them depends on several factors. When deciding upon a treatment modality, the location and dimension of the leakage point, leakage duration, the presence or absence of subfoveal fluid, and patient preference must be taken into account [9].

Continuous laser (CL) and micropulse laser (ML) are two of the most common types of laser therapy used in the treatment of CSCR. CL, also known as continuous-wave laser, photocoagulation laser, or thermal laser, is a high-intensity laser that is capable of delivering a continuous fascicle of light to target tissue, resulting in a visible burn and permanent scar [10]. CL is usually applied to extrafoveal or juxtafoveal leakage points as it can cause collateral damage to surrounding tissue, potentially leading to central scotoma, choroidal neovascularisation, or RPE atrophy [11]. CL is efficient in the reduction of SRF and improvement of visual acuity in CSCR; however, it has a relatively high rate of recurrence and side effects [12].

ML, also known as subthreshold laser, subvisible laser, sublethal laser, or tissue-sparing laser, is a low-intensity laser that delivers a series of short pulses of light to target tissue resulting in a subvisible and reversible effect [13]. ML is usually applied to areas of subfoveal or diffuse leakage, as it has been proven that it can result in photoreceptors and RPE sparing, resulting in the preservation of retinal function [14].

## Materials and methods

Study design

In this retrospective cohort study, we aimed to compare the efficacy of CL and ML therapy in the treatment of CSCR. Our approach focused on analyzing the primary outcome of reduction in average macular thickness (AMT), along with several secondary outcomes including changes in best corrected visual acuity (BCVA), subfoveal choroidal thickness (SFCT), and the resolution of SRF.

Ethical considerations were duly observed in accordance with the tenets of the Declaration of Helsinki. Due to its retrospective nature, the requirement for patient consent was waived. Nonetheless, all patient data were anonymized to maintain confidentiality.

Study population

The study included patients diagnosed with CSCR who had undergone treatment with either CL or ML at our institution. The inclusion criteria encompassed patients between 18 and 60 years of age, presenting with their first episode of CSCR, within one week of symptom onset, with active leakage on fluorescein angiography, SRF involving the fovea and related visual symptoms, documented treatment with CL or ML, and complete pre- and post-treatment records of BCVA and optical coherence tomography (OCT) imaging of the macula. Exclusion criteria encompassed patients with other retinal pathology, previous intraocular surgery, choroidal neovascularisation, anti-vascular endothelial growth factor (VEGF) injection treatment, and incomplete medical records.

Study protocol

All patients had an initial consultation and a follow-up at one-month post treatment. Initial consultation entailed fundus examination, OCT, and fundus fluorescein angiography (Topcon® Triton Plus Swept-Source DRI OCT, Oakland, New Jersey, United States) with fundus examination and OCT being repeated at the one-month follow-up. On each occasion, we noted AMT and SFCT (measured as the distance from the inner surface of the neurosensory retina to the inner surface of the choroid). The presence and resolution of SRF post treatment were also evaluated using OCT, with findings categorized into four distinct groups: constant, significantly less, significantly more, or resolved.

Interventions

The CL group received treatment with a 577-nm laser (Iridex 577® Laser System, Mountain View, California, United States) set on a 100 μm spot diameter, 100 ms duration, and power ranging from 50W to 70W. The endpoint of the treatment consisted of developing a slight white spot targeting the active leakage point previously identified on fluorescein angiography. 

The ML group parameters consisted of a 200 μm spot diameter, 200 ms duration, and a 5% duty cycle. Titration power ranged from 220W to 240W with threshold burn considered as a slight whitening reaction. Laser was applied on either macular leakage points or as a macular grid as a 4x4, 5x5, 6x6, or 7x7 array. Area of application overlaid diffuse leakage areas and areas of pigment epithelial detachment. 

Laser treatment was performed using the Volk Area Centralis lens (Mentor, Ohio, United States). 

Statistical analysis 

The primary outcome was a reduction in AMT, with secondary outcomes being BCVA improvement, SFCT reduction, and resolution of SRF at one month. For our statistical analysis, we employed independent t-tests to compare mean changes in continuous variables (AMT, AV, SFCT) between the CL and ML groups. The resolution of SRF, a categorical variable, was analyzed using the chi-squared test. Data were processed and analyzed using IBM SPSS Statistics for Windows, Version 26.0 (Released 2019; IBM Corp., Armonk, New York, United States), with a p-value of less than 0.05 considered statistically significant.

## Results

Patient demographics and baseline characteristics

The study encompassed a cohort of 121 patients (135 eyes) diagnosed with CSCR, who underwent treatment with either CL or ML. The demographics and baseline clinical characteristics were well matched across the two groups, as evidenced in Table 1, ensuring comparability for the treatment outcomes.

**Table 1:** Baseline demographics and clinical characteristics. CL: continuous laser; ML: micropulse laser; AMT: average macular thickness; BCVA: best corrected visual acuity; SFCT: subfoveal choroidal thickness; SD: standard deviation; *: t-test

Characteristic	CL group	ML group	P-value
Age, mean (SD), years	42.1 (5.7)	40.3 (6.1)	0.574^*^
Sex, %			
Male	69 (76%)	22 (24%)	0.476^*^
Female	21 (70%)	9 (30%)	
Eye			
OD	54	44	0.844^*^
OS	19	18	
Pre-treatment AMT, SD, 95% CI, μm	328.55 (60.90, 316.05-341.05)	306.85 (49.18, 290.16-323.55)	0.101^*^
Pre-treatment BCVA, SD, 95% CI, logMAR	0.31 (0.26, 0.26-0.36)	0.38 (0.25, 0.04, 0.29-0.47)	0.409^*^
Pre-treatment SFCT, SD, 95% CI, μm	436.94 (92.43, 12.70, 411.67-462.22)	474.63 (82.49, 15.88, 443.02-506.23)	0.121^*^

Primary outcome

Change in AMT

Our primary investigation focused on the change in AMT following treatment. In the CL group, the mean reduction in AMT was 51.14 µm, with a standard deviation (SD) of ±20. In contrast, the ML group exhibited a mean reduction of 29.88 µm, with an SD of ±22. This difference, however, did not reach statistical significance (p=0.10), indicating a similar effectiveness of both treatments in reducing AMT.

Secondary outcomes

BCVA

Improvement in visual acuity was observed in both groups. The CL group showed an average improvement in BCVA of 0.15 (±0.1), while the ML group demonstrated an improvement of 0.12 (±0.1). The difference between the groups was not statistically significant (p=0.41), suggesting that both treatments were comparably effective in improving visual acuity.

SFCT

The reduction in SFCT was more pronounced in the CL group, with a mean reduction of 32.19 µm (±15), compared to a mean reduction of 4.85 µm (±18) in the ML group. This difference was statistically significant (p=0.0004), highlighting the superior effectiveness of CL in reducing SFCT.

Resolution of SRF

Within the CL group, three eyes showed significantly increased SRF, and five eyes showed no change, while 20 eyes showed a significant reduction in SRF, and 40 eyes showed resolution of SRF. With regard to the ML group and SRF, two eyes displayed a significant increase, seven eyes displayed no change, 17 eyes displayed a significant decrease, and 13 eyes showed resolution. The post-treatment distribution of SRF changes did not differ significantly between the CL and ML groups, as indicated by a chi-squared test (p=0.065). This result suggests that both CL and ML have a similar effect on the resolution of SRF. 

Post-treatment values for both CL and ML groups are shown in Table 2.

**Table 2:** Post-treatment results and differences. CL: continuous laser; ML: micropulse laser; AMT: average macular thickness; BCVA: best corrected visual acuity; SFCT: subfoveal choroidal thickness; SD: standard deviation; *: t-test

Variable	CL group	ML group	P-value
Post-treatment AMT, SD, 95% CI, μm	277.41 (38.58, 269.49-285.33)	276.97 (17.06, 271.18-282.76)	0.9297^*^
Post-treatment BCVA, SD, 95% CI, logMAR	0.16 (0.23, 0.12-0.21)	0.26 (0.26, 0.17-0.34)	0.0718^*^
Post-treatment SFCT, SD, 95% CI, μm	398.15 (78.74, 376.41-419.90)	466.23 (79.71, 435.10-497.37)	0.0008^*^
AMT difference, SD, μm	51.14 (20)	29.88 (22)	0.10^*^
BCVA difference, SD, logMAR	0.15 (0.1)	0.12 (0.1)	0.4^*^
SFCT difference, SD, μm	32.19 (15)	4.85 (18)	0.0004^*^

## Discussion

Multiple studies looked at the efficacy and safety profile of CL and ML for CSCR, and the results have been summarised in a recent systematic review and meta-analysis that includes 15 randomized controlled trials [2]. The authors showed that ML was associated with a higher rate of complete SRF resolution, a lower rate of recurrence, and a better side effect profile when compared to CL. Additionally, ML was shown to have a similar or better effect on visual acuity, AMT, and SFCT. The review concluded that with regard to the management of CSCR, ML is superior to CL and recommends ML as a first-line treatment for CSCR.

The findings of our study contribute valuable insights into the comparative efficacy of CL and ML therapies in the treatment of CSCR. Our results demonstrate a notable difference in the reduction of SFCT between the two treatments, with CL showing greater efficacy in some aspects. This finding is particularly significant given the role of choroidal abnormalities in the pathogenesis of CSCR. However, both CL and ML were similarly effective in reducing AMT and improving visual acuity, underscoring the utility of both treatments in managing CSCR, which is in line with other retrospective studies [15].

The significant reduction in SFCT observed in the CL group aligns with the high-intensity nature of this therapy, which may induce a more pronounced tissue response. This aspect of CL treatment could be particularly beneficial in cases where choroidal involvement is more prominent, highlighting the importance of personalized treatment approaches based on individual patient characteristics and disease pathology. However, in our centre, patients who received ML therapy had leakage points close to the fovea or did not have clearly targetable focal leakage points, as applying CL to foveal or juxtafoveal leakage points is not safe [2]. In some situations, ML was also applied over focal RPE detachments. As such, given the retrospective nature of our study, patients who received ML therapy tend to be more complicated cases which may partly account for the difference in SFCT reduction. Furthermore, it is possible that longer follow-up may be warranted in order to assess long-term differences.

On the other hand, the lack of a significant difference in AMT reduction and BCVA improvement between CL and ML suggests that both therapies are effective in addressing these primary aspects of CSCR. This observation is consistent with the evolving literature on CSCR treatment, which increasingly emphasizes the need for individualized therapeutic strategies [2]. The choice between CL and ML should be based on specific patient needs, disease severity, location and potential to target leakage points, as well as the degree of choroidal involvement.

Recent studies have shown that in the treatment of CSCR, ML is at least as efficient as CL with regard to SRF resolution and visual acuity improvement, but has a smaller rate of recurrence and complications [16]. Our study's finding regarding the resolution of SRF post treatment, which showed no significant difference between the two treatment modalities, adds an important dimension to the ongoing discussion about the optimal management of CSCR. It suggests that both CL and ML can be effective options for patients, further supporting the notion of personalized treatment planning. Longer follow-up could potentially detect further improvements in the resolution of SRF [4,17].

The retrospective nature of our study and the inherent variability in patient responses to treatment underscore the complexity of CSCR management and the need for further research. Prospective, randomized controlled trials are needed to validate our findings and to explore the long-term efficacy and safety of CL and ML in the treatment of CSCR. Such studies would provide a more comprehensive understanding of the optimal treatment strategies for this condition and could lead to more effective management protocols.

## Conclusions

Our study highlights the effectiveness of both CL and ML in the treatment of CSCR, with specific advantages of CL in reducing SFCT. These findings contribute to the growing body of evidence in the field and underline the importance of considering individual patient characteristics and disease pathology in the selection of treatment modalities. Future research should focus on elucidating the long-term outcomes and potential differential effects of these laser therapies to optimize patient care in CSCR.
